# Metabolomic profiling and biological evaluation demonstrate the antioxidant, PPAR-γ, TAAR1, and FABP4 modulatory potential of *Strelitzia* species

**DOI:** 10.1038/s41598-026-37621-9

**Published:** 2026-02-18

**Authors:** Yasmine M. Rashad, Shaimaa Fayez, Rania F. Abou El-Ezz, Sherif A. Elsabbagh, Abdel Nasser B. Singab

**Affiliations:** 1 Department of Pharmacognosy, Faculty of Pharmacy, Misr International University, Cairo, Egypt; 2 Department of Pharmacognosy, Faculty of Pharmacy, Ain Shams University, Cairo, 11566 Egypt; 3 Department of Biochemistry, Faculty of Pharmacy, Galala University, Galala, Egypt; 4 Faculty of Pharmacy, Center for Drug Discovery Research and Development, Ain Shams University, Cairo, Egypt

**Keywords:** *Strelitzia* species, Antioxidant, GC-MS, ESI-MS/MS, PPAR-γ, TAAR1, FABP4, Molecular docking, Biochemistry, Cardiology, Diseases, Drug discovery

## Abstract

*Strelitzia* species, commonly known as the bird of paradise, are herbaceous perennial plants used as ornamentals owing to the beauty of their colored flowers. Due to lack of sufficient data on their chemical and pharmacological properties, the current study aimed to investigate the chemical profiles of two Egyptian *Strelitzia* species namely *S. reginae* Banks and *S. nicolai* Regel & Körn using multiplex of GC-MS and ESI-MS/MS metabolomics. Major components in the hexane extract of *S. reginae* Banks flowers were heneicosane, linoleic acid, and 17-octadecynoic acid whereas for *S. nicolai* Regel & Körn they were cumene, pseudocumene, hexahydrocumene, and propenyl cyclohexane. The hydrodistilled oil of *S. reginae* Banks flowers was enriched in caprylyl acetate, cembrene, caryophyllene, thunbergol, and verticilla-4(20),7,11-triene while for *S. nicolai* Regel & Körn they were heptacosane and 7-hexyleicosane. LC-MS/MS analysis of the methanol extracts of the leaves and flowers of both species revealed intra-and interspecies variations with general domination of phenolic acids, flavonoids, fatty acids, and phenolic derivatives. DPPH and FRAP analysis showed promising antioxidant activity for *S. reginae* Banks leaves. The hexane extract of *S. reginae* Banks showed agonistic activity to peroxisome proliferator activated receptor gamma (PPAR-γ) with EC_50_ value of 1.203 ± 0.045 µg/ml compared to rosiglitazone (EC_50_ = 0.292 ± 0.011 µg/ml), hinting at promising antidiabetic effect. Quantitative measurement of TAAR1 (trace amine associated receptor 1) levels in NCI-H810 cells treated with the hexane extract of *S. reginae* Banks flowers revealed remarkable decrease in its levels to 0.743 ± 0.025 ng/ml compared to untreated cells, hence showing premise for the treatment of depression and Parkinson disorders. The hexane extract of *S. reginae* Banks flowers moderately inhibited FABP4 (fatty acid binding protein 4) with IC_50_ value at 1.051 ± 0.028 µg/ml so it might be valuable for the management of hyperlipidemia and atherosclerosis. Our results were further supported by molecular docking studies to show the binding mechanisms of the natural ligands on their target proteins.

**Supplementary Information:**

The online version contains supplementary material available at 10.1038/s41598-026-37621-9.

## Introduction

 The Strelitziaceae is an ornamental family of flowering plants that belongs to order Zingiberales. The family includes three genera, of which *Strelitzia* is the most common, that disseminate in tropical and subtropical areas^1^. The Strelitziaceae was initially treated as a member of the Musaceae, however later it was separated as an independent family^1^. Genus *Strelitzia* is named after the birthplace of queen Charlotte (wife of George III) in Mecklenburg-Strelitz^1^. The genus is formed of five species including *S. reginae* Banks, *S. nicolai* Regel & Körn, *S. alba* (L.f.) Skeels, *S. caudata* R.A.Dyer, and *S. juncea* Andrews. The species (*S. reginae* Banks ex Aiton) is the most widely cultivated and is commonly known as the bird of paradise or the queen of paradise^1^. The plant is perennial with evergreen leaves and its flowers exist throughout the year, especially during the summer. The white bird of paradise or the wild banana (*Strelitzia nicolai* Regel & Körn) is native to South Africa with blue and white flowers and black seeds with orange arils^1^.

Unlike the other common plant pigments (i.e., carotenoids, anthocyanins, betalains, and chlorophylls), the vibrant bright orange color of the seed arils of *S. reginae* Banks and *S. nicolai* Regel & Körn is attributed to the presence of bilirubin-IX (an orange yellow tetrapyrrole and the end product of haem catabolism), which is the primary pigment responsible for the arils color to attract insects for pollination^2^. It was till 2009^3–5^ that bilirubin was thought to be exclusively present in mammals. Bilirubin is not just a catabolic product; however, it has been reported to display antioxidant and cytoprotective effects against cancer, diabetes, cardiovascular diseases, and neurological disorders^6^. Each flower of *S. reginae* Banks is formed of three orange sepals and three blue petals. The orange and bluish colors of the flowers are attributed to the carotenoids (at least 19 were discovered) and anthocyanins (mainly delphinidin-3-rutinoside), respectively^5,7^.

Despite the commercial value of the bird of paradise, there is a paucity of knowledge in literature concerning the phytochemical and biological effects of *Strelitizia* species. A study by Ali et al.^8^ revealed a promising in vivo anti-arthritic potential of the methanol aerial extract by reducing paw edema, reducing interleukin-17a, THF-α, interleukin-1β, receptor activator of nuclear factor kappa-beta ligand, and interferon-γ. Another study showed that the ethyl acetate and butanol fractions of *S. reginae* Banks displayed good antimicrobial activity against *Klebsiella pneumonia* and *Staphylococcus aureus*, yet they were inactive against *Pseudomonas aeruginosa* and *Candida albicans* clinical isolates^9^.

Phenalenones such as anigorufone and its hydroxy derivative, 8-hydroxy-7 methoxy-6-phenylphenalen-1-one, and 5-hydroxy 6-methoxy-7-phenylphenalen-1-one were isolated from the hexane and chloroform extracts of *S. reginae* Banks rhizomes^10^. These phenalenones have previously been reported from species belonging to family Musaceae confirming the close chemotaxonomic relationship between the two families^10^. Flavonoids such as kaempferol, quercetin, vitexin, and rutin have been isolated from the leaves of *S. reginae* Banks^9^. Plants enriched with flavonoids are known to display strong antioxidant power^11–13^.

In the current study, we performed detailed chemical profiling of *S. reginae* Banks and *S. nicolai* Regel & Körn polar and non-polar extracts using ESI-MS/MS and GC-MS, respectively. The extracts were likewise tested for their antimicrobial, antioxidant, anti-inflammatory, PPAR-γ agonistic, TAAR1 and FABP4 inhibitory activities. Biological data were further supported by molecular docking approaches.

## Materials and methods

### Plant collection and extraction

Fresh leaves and flowers of *S. reginae* Banks and *S. nicolai* Regel & Körn were collected from Orman botanical garden in Cairo. Authentication of the plants were kindly performed by Taxonomy Specialist Terase Labib, Consultant of Plant Taxonomy at the Ministry of Agriculture, and El-Orman Botanical Garden, Giza, Egypt. The flowers of *S. reginae* Banks (15 flower weighing ca. 400 g) and *S. nicolai* Regel & Körn (1 flower weighing 400 g) were hydrodistilled on a Clevenger system for 5 h to yield 0.2 ml of *S. reginae* Banks and one drop of *S. nicolai* Regel & Körn oil. For hexane extraction, 250 g and 200 g of dried and milled *S. reginae* Banks and *S. nicolai* Regel & Körn flowers, respectively, were cold macerated in (3 × 1 l) hexane. The extract was filtered and concentrated before GC-MS analysis. For LC-MS measurements, dried powdered leaves and flowers of *S. reginae* Banks (10 g leaves, 5 g flowers) and *S. nicolai* Regel & Körn (5 g leaves, 5 g flowers) were macerated till exhaustion in methanol to yield 0.3 g and 0.45 g crude extract of *S. reginae* Banks flowers and defatted leaves, respectively, as well as 0.35 g and 0.22 g of *S. nicolai* Regel & Körn flowers and defatted leaves, respectively.

### GC-MS analysis of hexane extracts and hydrodistilled oils of *S. reginae* Banks and *S. nicolai* Regel & Körn flowers

GC-MS measurements were performed following similar previous protocols^14,15^ on a Shimadzu QP-2010 (Shimadzu, Kyoto, Japan) coupled to a quadrupole mass spectrometer. Chromatographic separation was performed on Rtx-5MS (30 m × 0.25 mm i.d. × 0.25 μm) capillary column (Restek, Bellefonte, PA, USA) in a split injection mode with a split ratio of 1:15. Initial column temperature was set at 45 ℃ for 2 min then gradually increased at a rate of 5 ℃/min till reaching 300 ℃. Helium was used as the carrier gas flowing at a rate of 1.41 ml/min. Samples were injected (injection volume of 1 ml) after being diluted to 1% v/v. Oven and injector temperatures were adjusted to 50 ℃ and 250 ℃, respectively. Ionization energy was set to 70 eV with a scan range at *m/z* 35–600. Identification of components was based on a comparison of MS^2^ fragments with those present in an in-house NIST-17 library. Retention indices (Kovat’s indices) were calculated after injecting a series of *n*-alkanes (C_8_–C_30_) under the same GC-MS conditions then compared to those reported in NIST chemistry webbook online database and those previously reported in the literature.

### LC-ESI-MS/MS measurements

LC-MS measurements were performed as described in our previous reports^16^. Briefly, A XEVO TQD triple quadruple mass spectrometer (Waters, Milford, USA) equipped with ACQUITY UPLC-BEH C_18_ column (1.7 μm, 2.1 × 50 mm) was used with a flow rate of 0.2 ml/min. A gradient elution system was applied using water in 0.1% formic acid as solvent A and methanol as solvent B as follows: 10–30% B (0–5 min), 30–70% (5–15 min), and 70–90% (15–25 min). Mobile phases were degassed at 25 °C for 15 min. Samples were filtered and the injection volume was set to 4 µl. Mass acquisition was acquired in positive and negative modes with *m/z* scan range 100–1300 amu, a capillary voltage of 3 kV, ramped collision energies (20 V for *m/z* 100–300, 25 V for *m/z* 300–400, 30 V for *m/z* 400–500, 35 V for *m/z* 500–700, 40 V for *m/z* 700–1000, 50 V for *m/z* > 1000), ion source temperature 150 ℃, desolvation temperature 400 ℃, cone gas flow at 50 l/h, and desolvation gas flow at 600 l/h. Data acquisition was performed using Waters MassLynx SCN 940 (https://www.waters.com).

### Molecular docking

#### Target prediction

The three-dimensional structures of FABP4 (2NNQ), TAAR1 (8W89), and PPAR**-γ** (8DK4) in complex with their respective inhibitors were retrieved from the protein data bank (https://www.rcsb.org/). Protein structures were prepared by removing water, impurities, and the bound ligand using PyMOL (https://pymol.org/2/). The missing atoms, residues, and hydrogen atoms (appropriate for pH 7.4) were added using PDBFixer tool^17^.

#### Ligand preparation

Structures of the major components identified in the GC-MS analysis of the hexane extracts of *S. reginae* Banks and *S. nicolai* Regel & Körn flowers were downloaded from PubChem database in 3D SDF format. Using openbabel 3.0 software (https://openbabel.org/index.html), hydrogen atoms (suitable for pH 7.4) were added, and energy was minimized using MMFF94s forcefield.

#### Virtual screening

Molecular docking was performed using Smina (https://sourceforge.net/projects/smina/), a fork of autodock vina^18^ that has several built-in scoring functions estimating the protein-ligand binding affinity. Docking experiment was validated by re-docking the co-crystallized ligands into the respective protein active site using the various scoring functions. RMSD values between the experimental and the redocked poses were calculated and the scoring function resulting in the lowest RMSD was used for docking procedure. Subsequently, the natural compounds were docked in place of the bound inhibitor accommodating the active site and the generated poses were ranked based on the docking scores. Protein-Ligand Interaction Profiler (PLIP) server (https://plip-tool.biotec.tu-dresden.de/plip-web/plip/index) was used to analyze the interactions between the top hit compounds and the target proteins^19^. Protein-ligand complexes were visualized using PyMOL.

### Antimicrobial assays

#### Preparing inoculum (colony suspension method)

The antimicrobial assay was performed following the previous protocol reported by Mahran et al.^20^. In brief, a disc with each organism was inoculated into 100 ml of tryptic soy broth medium and incubated at 37 °C for 24 h except for *Bacillus cereus* which was incubated at 30.0 ℃ for 24 h. For the fresh culture, a loopful from the broth was streaked onto tryptic soy agar then incubated for 18–24 h at the same previous temperature. A sterile saline solution was prepared by inoculating 3–4 colonies from the organism and the suspension was adjusted to achieve a turbidity equivalent to 0.5 McFarland standard of each strain using DensiCHEK© optical device which results in a suspension containing ca. 1–2 × 10^8^ CFU/ml. The suspension was diluted by inoculating 1 ml of the inoculum into 150 ml of Muller Hinton broth, resulting in approximately a concentration of 1 × 10^6^ CFU/ml. Any subsequent 1:2 dilution shall result in 5 × 10^5^ CFU/well.

#### Broth microdilution method

1 ml of Muller Hinton broth (MHB) was inoculated into 24 wells plate. 2 ml from each sample was inoculated in the first well, then 1 ml was aspirated and transferred to the next well, previously filled with 1 ml MHB, to make 1:2 dilution. They were properly mixed then 1.0 ml was aspirated and added to the next 1 ml broth (1:4 dilution). The latter step was repeated for preparing at least 8 dilutions where 1 ml of the prepared inoculum was added to each well resulting in a final concentration of 5 × 10^5^ CFU/well (the recommended concentration is 2–8 × 10^5^ CFU/ml). A 1 ml from organism suspension was diluted and cultured externally to confirm inoculum density. A negative control well containing only the broth without sample nor bacteria was added to each sample plate. All plates were incubated at 37 °C for 24 h except for *Bacillus cereus* which was incubated at 30 °C for 24 h. After incubation, plates were removed from the incubator and placed in the dark to check the growth. All growth control wells yielded turbid solution while all negative control wells were found clear, indicating validity of the test. Inoculum density culture results had a concentration of 4–6 × 10^5^ CFU/ml for the tested organism. The MIC of each extract was defined as the lowest concentration that completely inhibited bacterial growth after 24 h of incubation.

### In vitro antioxidant assays

#### DPPH free radical assay

The DPPH (2,2-diphenyl-1-picryl-hydrazyl-hydrate) free radical assay was carried out according to the method of Boly et al.^21^. Briefly, 100 µl of freshly prepared DPPH (0.1% in methanol) was added to 100 µl of each extract in 96 wells plate (*n* = 6). The reaction was incubated at room temperature for 30 min in the dark. After incubation, the resulting reduction in DPPH color was measured at 540 nm. Data are represented as mean ± SD according to the following equation:

% inhibition = [(Average absorbance of blank - average absorbance of the test)/(Average absorbance of blank)] × 100.

The results were recorded using the microplate reader FluoStar Omega. Data was analyzed using Microsoft Excel^®^ and the IC_50_ values were calculated using Graph pad Prism 6^®^ by converting the concentrations to their logarithmic value and selecting non-linear inhibitor regression equation (log (inhibitor) vs. normalized response – variable slope equation).

#### FRAP (ferric reducing antioxidant power) assay

The assay was carried out according to the method of Benzie et al.^22^ with minor modifications. A freshly prepared TPTZ reagent (300 mM acetate Buffer at pH of 3.6, 10 mM TPTZ in 40 mM HCl, and 20 mM FeCl_3_, in a ratio of 10:1:1 v/v/v, respectively). 190 ul from the freshly prepared TPTZ were mixed with 10 ul of the sample in 96 wells plate (*n* = 3), then the reaction was incubated at room temperature for 30 min in dark. After incubation, the resulting blue color was measured at 593 nm using microplate reader FluoStar Omega and data was represented as mean ± SD. The ferric reducing ability of the samples is presented as µM TE/mg sample using the linear regression equation extracted from the linear dose-response curve of trolox.

#### ABTS (2,2-azino-bis-3-ethylbenzothiazoline-6-sulphonic acid) assay

The assay was carried out according to the method of Arnao et al.^23^ with minor modifications. Briefly, 192 mg of ABTS reagent was dissolved in distilled water and transferred to 50 ml volumetric flask then the volume was completed with distilled water. 1 ml of the solution was added to 17 µl of 140 mM potassium persulphate and the mixture was left in the dark for 24 h. About 1 ml of the reaction mixture was completed to 50 ml methanol to obtain the final ABTS dilution used in the assay. 190 µl of ABTS was mixed with 10 µl of the sample (100 µg/ml of the methanol extract of *S. reginae* Banks leaves) in 96 wells plate (*n* = 6) and the reaction was incubated at room temp. for 30 min in the dark. After incubation, the decrease in ABTS color was measured at 734 nm using microplate reader FluoStar Omega. Data are represented as mean ± SD according to the following equation:

% inhibition = [(Average absorbance of blank - average absorbance of the test)/(Average absorbance of blank)] × 100.

#### ORAC (oxygen radical absorbance capacity) assay

The assay was carried out according to the method of Liang et al.^24^ with few modifications. Briefly, 10 µl of the sample (50 µg/ml of the methanol extract of *S. reginae* Banks leaves) was incubated with 30 µl fluoresceine (100 nM) for 10 min at 37 ℃. Fluorescence measurement (at 485 nm excitation and 520 nm emission) was carried out using microplate reader FluoStar Omega for three cycles (90 s. each) for background then 70 µl of 300 mM freshly prepared 2,2’-Azobis(2-amidinopropane) dihydrochloride (AAPH) was added immediately to each well. Fluorescence measurement (at 485 nm excitation and 520 nm emission) was continued for 60 min (40 cycles, 90 s. each). Data are represented as means ± SD (*n* = 3) and the antioxidant effect of the extract was calculated as µM trolox equivalents by substitution in the linear regression equation extracted from standard trolox calibration curve.

#### Ferrozine iron metal chelation assay

The assay was carried out according to the method of Santos et al.^25^ with minor modifications to be carried out in microplates. Briefly, 20 µl of the freshly prepared ferrous sulphate (0.3 mM in acetate buffer at pH 6) were mixed with 50 µl of 1 mg/ml sample (the methanol extract of *S. reginae* Banks leaves) in 96 wells plate and 50 µl acetate buffer (*n* = 6). 30 µl of ferrozine (0.8 mM in acetate buffer) was added to each well and the reaction mixture was incubated at room temperature for 10 min. After incubation, the decrease in the produced color was measured at 562 nm using microplate reader FluoStar Omega. Results are presented as µM EDTA equivalent/mg sample using the linear regression equation extracted from EDTA calibration curve. Data are represented as mean ± SD according to the following equation:

% inhibition = [(Average absorbance of blank - average absorbance of the test)/(Average absorbance of blank)] × 100.

### Anti-inflammatory assay

#### Cell culture

RAW 264.7 mouse macrophage cell line was obtained from Nawah Scientific Inc., (Mokatam, Cairo, Egypt). Cells were maintained in DMEM media supplemented with 100 mg/ml streptomycin, 100 units/ml penicillin and 10% of heat-inactivated fetal bovine serum in humidified, 5% (v/v) CO_2_ atmosphere at 37 °C.

#### In vitro anti-inflammatory assay

RAW264.7 cells were seeded into a 96-well plate and incubated for 24 h. Inflammation was induced with 1 µg/ml lipopolysaccharide (LPS-group) and untreated cells was replenished with fresh media (control group). *Strelitzia* extracts were treated with LPS in two/five concentrations (LPS + extract). Dexamethasone (1 μm) was used as an anti-inflammatory positive control. To measure nitric oxide (NO) secretion, equal volumes of the cell supernatant and Griess reagent were mixed for 10 min in the dark at room temperature. The absorbance at 540 nm, representing the nitrite concentration, was measured using an ELISA plate reader.

#### Cytotoxicity assay

Cell viability was assessed by SRB assay which was performed using a similar protocol previously reported^26^ with minor modifications. Aliquots of 100 µl cell suspension (5 × 10^3^ cells) were in 96-well plates and incubated in complete media for 24 h. Cells were treated with another aliquot of 100 µl media containing the tested extracts at various concentrations. After 72 h of sample exposure, the cells were fixed by replacing media with 150 µl of 10% TCA and incubated at 4 °C for 1 h. The TCA solution was removed and the cells were washed 5 times with distilled water. Aliquots of 70 µl of 0.4% w/v SRB solution were added and incubated in a dark place at room temperature for 10 min. Plates were washed 3 times with 1% acetic acid and allowed to air-dry overnight. Then, 150 µl of TRIS (10 mM) was added to dissolve protein-bound SRB stain and the absorbance was measured at 540 nm using a BMG LABTECH^®^- FLUOstar Omega microplate reader (Ortenberg, Germany).

### PPAR-γ luciferase reporter assay

The PPAR-γ activity was measured using a reporter assay kit (Indigo Biosciences, PA, USA) according to the manufacturer’s instructions and as previously reported in the literature^27^ with some modifications. The reporter cells (provided with the assay kit) are non-human mammalian cells that are steadily transfected with human PPAR-γ and beetle luciferase reporter genes. The cells are dispensed into 96-well plate and are cultured in a cell recovery medium (CRM) for 4 h then subsequently treated with 10 µg/ml of the hexane extracts of *S. reginae* Banks and *S. nicolai* Regel & Körn flowers (dissolved in the compound screening medium CSM provided by the supplier) and the reference agonist rosiglitazone. Control cells were treated with CSM only. After 48 h incubation, the treatment medium was discarded, and the luciferase detection reagent (LDR) (Indigo Biosciences, PA, USA) was added. Intensity of light emission from each well is quantified using Bioline™ Elisa plate reader at 450 nm to determine PPAR-γ agonistic activity. The half effective concentration (EC_50_) values on luciferase activity were analyzed using GraphPad Prism (GraphPad Software Inc., La Jolla, CA).

### Human trace amine associated receptor-1 (TAAR1) immunoassay

The assay was performed using a human trace amine-associated receptor 1 ELISA Kit (catalog#: MBS1608896) according to the manufacturer’s instructions. In brief, NCI-H810 cells were cultured in Dulbecco’s Modified Eagle Serum (DMEM), supplemented with 10% fetal bovine serum, and maintained at 37 ℃ with 5% CO_2_. The cells were seeded in 96-well plate and incubated for 24 h prior to treatment with the sample (10 µg/ml of the hexane extract of *S. nicolai* Regel & Körn flowers dissolved in DMSO). After 48 h incubation, cells were destructed through repeated freeze-thaw cycles to let out the inside components then centrifuged at 2000 rpm for 20 min and the supernatant was collected. 50 µl standard was added to the standard well and 40 µl sample (cell supernatant) was added to the sample well then 10 µl anti-TAAR1 antibody was added to sample well. 50 µl streptavidin-HRP was added to sample and standard wells. After mixing, the plate was covered with a sealer and incubated for 60 min at 37 ℃. After washing the plates, 50 µl of substrate solution A and substrate solution B were added to each well then incubated for 10 min at 37 ℃. 50 µl of stop solution was added to each well and the blue color changed into yellow immediately. Within 10 min, the optical density (OD) value of each well was recorded using ROBONIK P2000 ELISA reader at 450 nm.

### Fatty acid binding protein 4 (FABP4) screening assay

The assay was performed using a FABP4 screening/ligand assay kit (Cayman Chemical Company, Michigan, USA) according to the manufacturer protocol and as previously reported in the literature^28,29^ with few modifications. The kit is composed of arachidonic acid control, FABP assay buffer, FABP assay detection reagent, and FABP (human recombinant) assay reagent. 40 µl of FABP assay buffer is added to 25 µl of FABP4 protein and 25 µl of detection reagent in each well. Then 10 µl of the tested sample (10 µg/ml of the hexane extract of *S. reginae* Banks flowers dissolved in DMSO) and/or the positive control (cobimetininb) were added. The plate was covered and incubated at room temperature for 10 min., then the fluorescence was measured using a Tecan-spark^®^ reader at 450 nm after excitation at 360 nm.

### Statistical analysis

The results were represented as mean ± SD. Statistical significance was computed by applying one-way analysis of variance (ANOVA) followed by Dunnett’s test for multiple comparisons. Unpaired t-test was used to compare one treatment group with the control. *p* values < 0.05 were considered as statistically significant. GraphPad Prism software, version 9.00 (GraphPad Software, Inc., La Jolla, CA, USA) (https://www.graphpad.com) was used for data analysis and graph plotting.

## Results and discussion

### GC-MS analysis of the hexane extracts and hydrodistilled oils of *Strelitzia reginae* Banks and *Strelitzia nicolai* Regel & Körn flowers

**Fig. 1:**
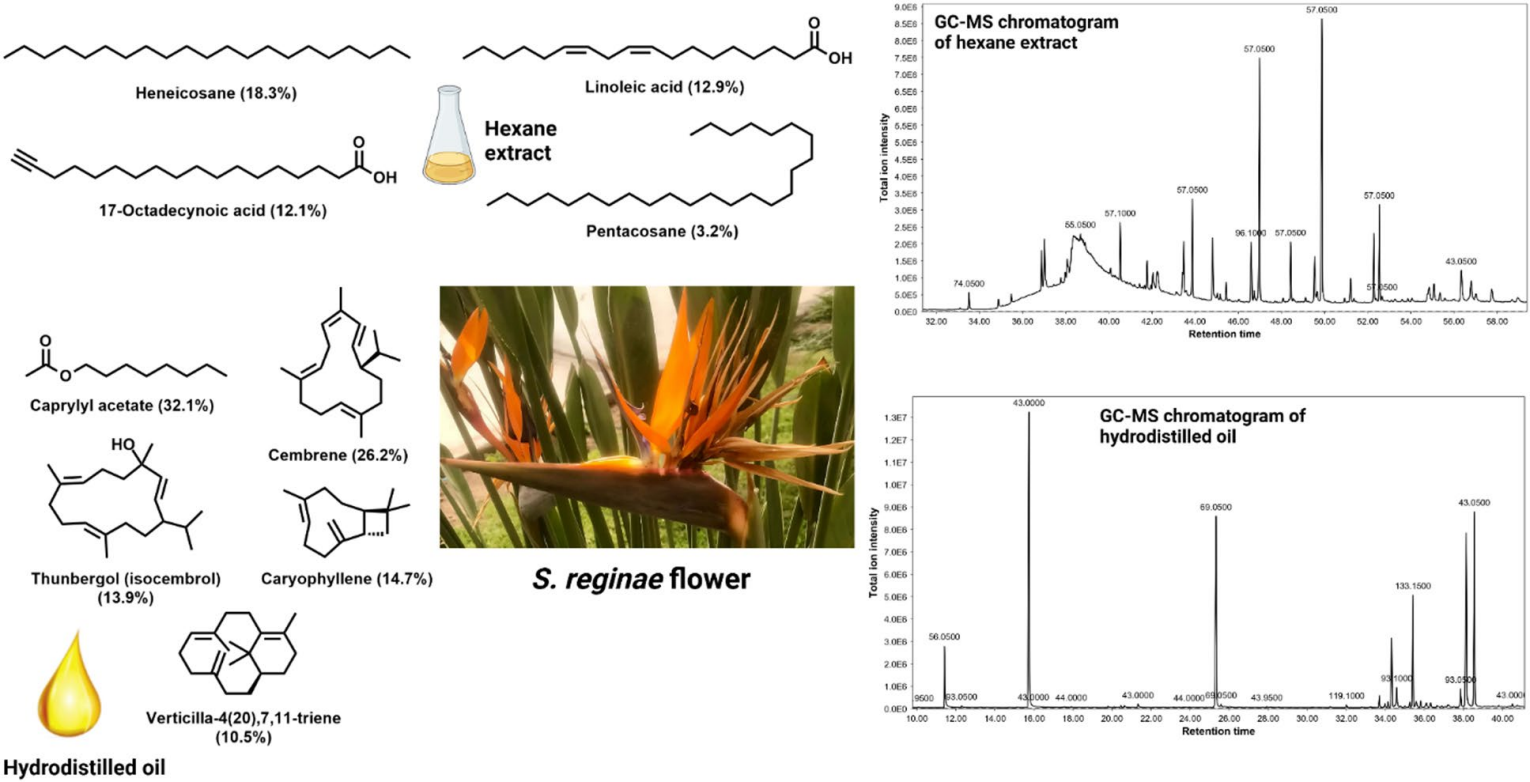
The GC-MS chromatograms and the structures of the major components identified in the hexane extract and hydrodistilled oil of *S. reginae* Banks flowers.

The hexane extracts and the hydrodistilled oils obtained from the flowers of *S. reginae* Banks and *S. nicolai* Regel & Körn were subjected to GC-MS for analysis of their chemical components (Table 1). The hexane extract of *S. reginae* Banks flowers showed predominance of hydrocarbons and fatty acids which represented 29.6% and 27.3% of the total chromatogram area, respectively. The major hydrocarbon was heneicosane which constituted 18.3%, whereas the main fatty acids were linoleic (12.9%) and 17-octadecynoic (12.1%) acids (Fig. 1). Fatty acids esters and fatty alcohols showed comparable percentages representing 6.9% and 6.8% of the total GC-MS chromatogram, respectively. Esters, other than fatty acid esters, such as ambrettolide, bis(2-ethylhexyl)phthalate, and 14-tricosenyl formate constituted ca. 6% of the total identified compounds. Phthalate esters were detected in higher concentrations in the hexane extracts of *S. reginae* Banks (2.5%) and *S. nicolai* Regel & Körn (2.8%), yet they were minor in the oil of *S. nicolai* Regel & Körn (0.36%) and totally absent in the oil of *S. reginae* Banks. Sterols and their esters were likewise detected in the hexane extract of *S. reginae* Banks flowers, and they represented about 3.9% of the total GC-MS chromatogram. The hydrodistilled oil of *S. reginae* Banks flowers is dominated with diterpenes, fatty acid esters, diterpene alcohols, and sesquiterpenes. The only fatty acid ester detected was caprylyl acetate which alone represented 32.1% of the total extract. Similarly, caryophyllene was the sole sesquiterpene present in the oil and constituted 14.7% of the GC-MS chromatogram. The diterpenes were represented by cembrene (26.2%), verticilla-4(20),7,11-triene (10.5%), and isoneocembrene A (0.3%), whereas diterpene alcohols were represented by thunbergol (13.9%) and verticiol (1.76%).

**Fig. 2:**
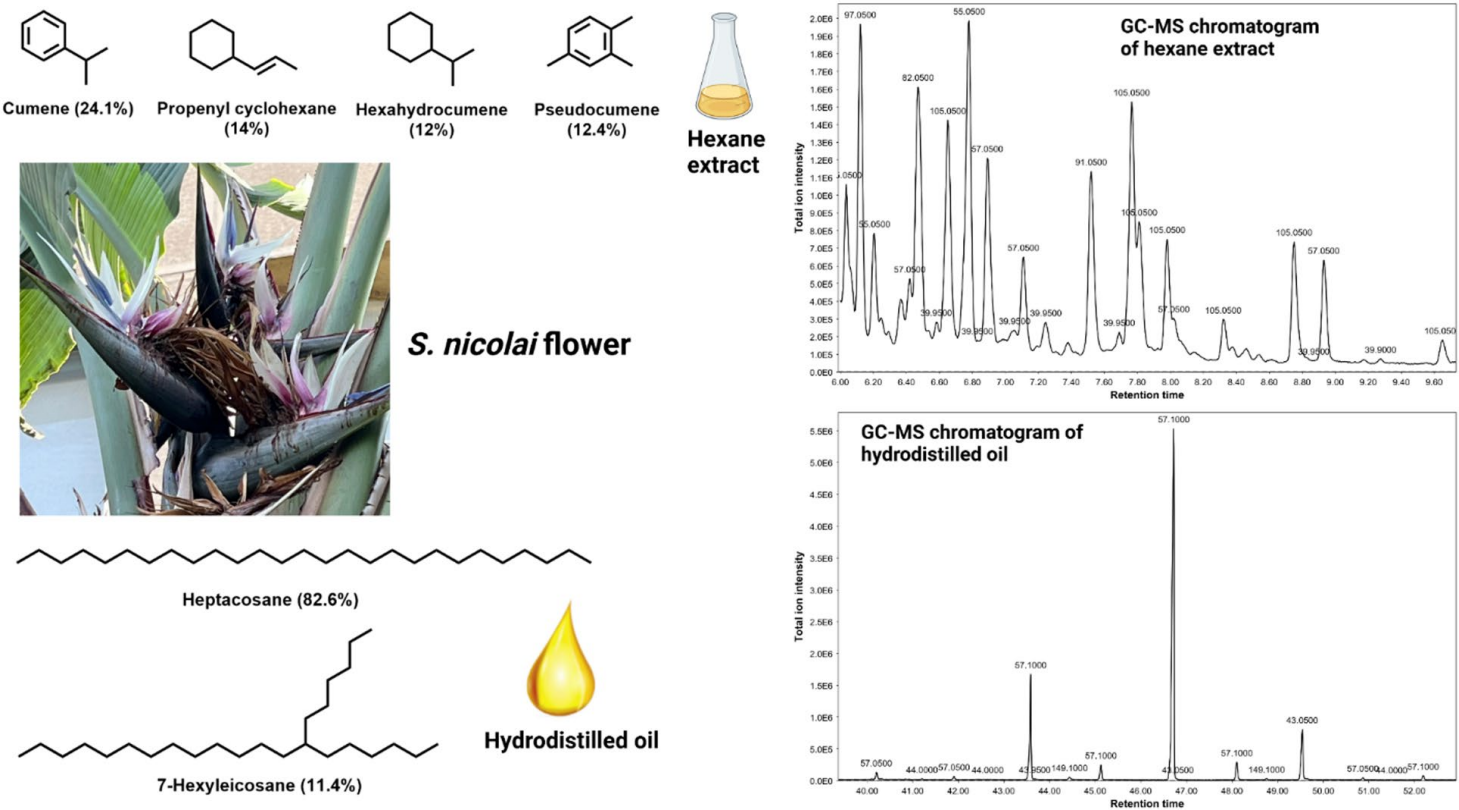
The GC-MS chromatograms and the structures of the major components identified in the hexane extract and hydrodistilled oil of *S. nicolai* Regel & Körn flowers.

GC-MS investigations on the hexane extract of *S. nicolai* Regel & Körn flowers showed the predominance of hydrocarbons which represent nearly half of the GC-MS chromatogram with propenylcyclohexane, hexahydrocumene, 1-ethyl-3-methylcyclohexane, and 3-methylnonane representing 14%, 12%, 11.3%, and 6.9% of the total extract, respectively. Aromatic hydrocarbons constituted ca. 43.3% of the GC-MS chromatogram and were presented by cumene (24.1%), pseudocumene (12.4%), and isocumene (6.8%) (Fig. 2). It is noteworthy to hint that there were no components in common between the hexane extracts of *S. reginae* Banks and *S. nicolai* Regel & Körn except for the phthalate ester mentioned previously. The hydrodistilled oil of *S. reginae* Banks is exclusively dominated by hydrocarbons constituting 98.8% with heptacosane being the most abundant alkane (82.6%) followed by 7-hexyleicosane (11.4%) (Fig. 2). The richness of the essential oils of *S. reginae* Banks in diterpenoids and *S. nicolai* Regel & Körn in saturated alkanes is responsible for the lack of aroma in these flowers despite their fascinating inflorescence. This was further validated by headspace GC-MS analysis of freshly obtained flowers where the chromatograms showed almost no peaks and the total yield of the hydrodistilled oils which was extremely low. As for the best of our knowledge, the current study is the first to study the chemical composition of the oils obtained from *S. reginae* Banks and *S. nicolai* Regel & Körn flowers by hydrodistillation and hexane extraction. A single study had previously been conducted^30^ on the hydrodistilled oil obtained from the seed arils of *S. nicolai* Regel & Körn showing the predominance of amines (31.7%) and ethers (28.1%) which were both absent in our studied samples.

**Table 1:** GC-MS analysis of the hydrodistilled and hexane-extracted oils of the flowers of the two Strelitzia species, *S. reginae* banks and *S. nicolai* Regel & Körn.

No.	t_R_ (min)	Annotated compound	KI_exp_	KI_rep_	Formula	Chemical class	Relative area (%)				SI
							*S. reginae*		*S. nicolai*		
							Hexane	Oil	Hexane	Oil	
1	6.1	1-Ethyl-3-methylcyclohexane	902	894	C_9_H_18_	Hydrocarbons	×	×	11.3	×	95
2	6.4	Propenylcyclohexane	914	923.3	C_9_H_18_O		×	×	14.0	×	87
3	6.7	Hexahydrocumene	924	924	C_9_H_18_		×	×	12.0	×	94
4	6.8	3-Methylnonane	928	951	C_10_H_22_		×	×	6.9	×	92
5	7.1	2-Ethyl-4-methylpentanol	936	931	C_8_H_18_O	Aliphatic alcohol	×	×	4.4	×	91
6	7.2	3-Methyl-1-cyclooctene	940	958	C_9_H_16_	Hydrocarbon	×	×	1.2	×	90
7	7.5	Isocumene	949	950	C_9_H_12_	Aromatic hydrocarbons	×	×	6.8	×	80
8	7.7	Cumene	958	958	C_9_H_12_		×	×	24.1	×	94
9	7.9	Pseudocumene	965	976	C_9_H_12_		×	×	12.4	×	96
10	8.4	Menthane	982	982	C_10_H_20_	Monoterpene	×	×	0.4	×	89
11	8.9	Decane	998	1000	C_10_H_22_	Hydrocarbon	×	×	3.3	×	95
12	15.8	Caprylyl acetate	1201	1202	C_10_H_20_O_2_	Fatty acid ester	×	32.1	×	×	97
13	25.4	Caryophyllene	1551	1555	C_15_H_24_	Sesquiterpene hydrocarbon	×	14.7	×	×	87
14	33.5	Methyl palmitate	1905	1904	C_17_H_34_O_2_	Fatty acid ester	0.61	×	×	×	96
15	33.8	Cembrene (Thunbergen)	1921	1923	C_20_H_32_	Diterpene	×	26.2	×	×	92
16	34.8	Ethyl palmitate	1971	1975	C_18_H_36_O_2_	Fatty acid ester	0.31	×	×	×	96
17	35.4	Isoneocembrene A	1996	2019	C_20_H_32_	Diterpene	×	0.30	×	×	81
18	35.4	Isopropyl palmitate	1999	1999	C_19_H_38_O_2_	Fatty acid ester	0.30	×	×	×	94
19	35.5	Verticilla-4(20),7,11-triene	2004	2004	C_20_H_32_	Diterpene	×	10.5	×	×	93
20	36.4	Verticiol	2054	2036	C_20_H_34_O	Diterpene alcohol	×	1.76	×	×	83
21	36.8	9,12-Octadecenoic acid, methyl ester	2076	2076	C_19_H_34_O_2_	Fatty acid esters	1.37	×	×	×	91
22	37.0	Linolenic acid, methyl ester	2083	2082	C_19_H_32_O_2_		2.33	×	×	×	93
23	37.7	Isobutyl palmitate	2125	2135	C_20_H_40_O_2_		0.28	×	×	×	94
24	38.0	*cis*−8,11,14-Eicosatrienoic acid	2141	2135	C_20_H_34_O_2_	Fatty acids	2.37	×	×	×	88
25	38.3	17-Octadecynoic acid	2158	2165	C_18_H_32_O_2_		12.1	×	×	×	82
26	38.6	Linoleic acid	2175	2173	C_18_H_32_O_2_		12.9	×	×	×	87
27	38.7	Thunbergol	2177	2157	C_20_H_34_O	Diterpene alcohol	×	13.9	×	×	74
28	40.0	Eicosan-1-ol	2252	2252	C_20_H_42_O	Fatty alcohol	1.71	×	×	×	96
29	40.3	Musk ambrette (Ambrettolide)	2264	2246	C_16_H_28_O_2_	Lactone	0.97	×	×	×	76
30	40.5	Heneicosane	2276	2109	C_21_H_44_	Hydrocarbons	1.83	×	×	×	98
31	41.9	7-Methylpentadecane	2387	n.d	C_16_H_34_		×	×	×	0.49	95
32	42.0	Eicosatetraenoic acid, methyl ester	2359	2308	C_21_H_34_O_2_	Fatty acid ester	0.79	×	×	×	88
33	43.4	1-Heneicosanol	2444	2402	C_21_H_44_O	Fatty alcohol	2.51	×	×	×	95
34	43.5	1-Pentacosene	2451	2486	C_25_H_50_	Hydrocarbons	0.43	×	×	×	81
35	43.8	Pentacosane	2470	2483	C_25_H_52_		3.20	×	×	×	96
36	44.4	Bis(2-ethylhexyl) phthalate	2542	2544	C_24_H_38_O_4_	Ester	2.50	×	2.88	0.36	98
37	45.0	Oleyl acetate	2544	2185	C_20_H_38_O_2_		0.36	×	×	×	91
38	45.1	7-Hexyleicosane	2586	2542	C_26_H_54_	Hydrocarbon	0.60	×	×	11.4	96
39	46.5	(Z)−14-Tricosenyl formate	2644	2679	C_24_H_46_O_2_	Ester	2.56	×	×	×	90
40	46.7	Heptacosane	2690	2700	C_27_H_56_	Hydrocarbon	×	×	×	82.6	95
41	46.7	Undec-10-ynoic acid, tetradecyl ester	2654	2671	C_18_H_34_O	Fatty ester	0.54	×	×	×	89
42	46.9	Heneicosane	2670	2109	C_21_H_44_	Hydrocarbons	18.37	×	×	×	96
43	48.1	Octacosane	2784	2800	C_28_H_58_		1.96	×	×	4.39	97
44	49.5	1-Nonacosene	2834	2894	C_29_H_58_		2.24	×	×	×	90
45	51.2	Tetratetracontane	2941	n.d	C_44_H_90_		0.79	×	×	×	96
46	52.2	16-Hentriacontanol	3010	3265	C_31_H_64_O	Fatty alcohol	2.40	×	×	×	93
47	52.5	Tetratriacontane	3027	3401	C_34_H_7_0	Hydrocarbon	2.94	×	×	×	96
48	52.6	Octacosanol	3035	3047	C_28_H_58_O	Fatty alcohol	0.26	×	×	×	89
49	54.8	Ergost-5-en-3-ol	3175	3165	C_28_H_48_O	Sterol	1.25	×	×	×	84
50	55.3	Hexatriacontane	3207	3600	C_36_H_74_	Hydrocarbon	0.46	×	×	×	89
51	56.3	β-Sitosterol acetate	3270	3357	C_31_H_52_O_2_	Sterols	1.91	×	×	×	87
52	57.7	Cycloartenol acetate	3361	3389	C_30_H_50_O		0.78	×	×	×	85
(%) Total identified compounds							83.9%	99.4%	99.6%	99.2%	

### LC-MS/MS analysis of the methanol extracts of the leaves and flowers of *S. reginae* Banks and *S. nicolai* Regel & Körn

**Fig. 3:**
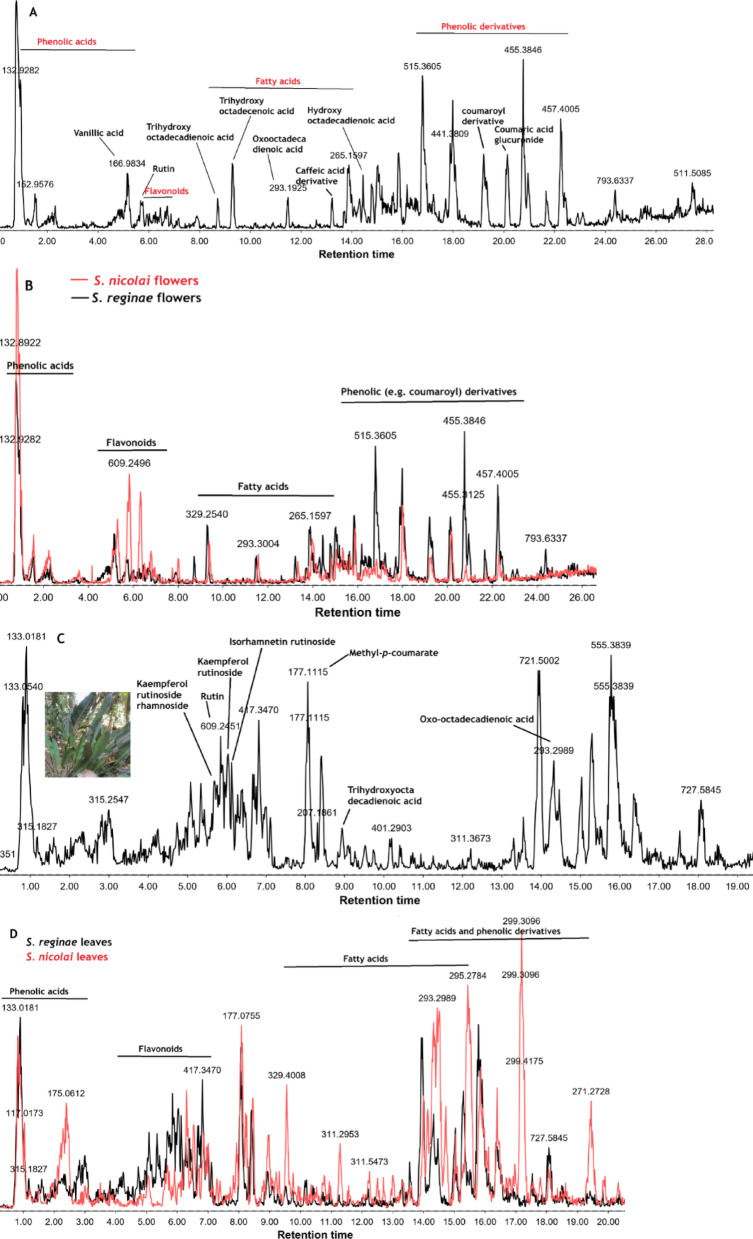
(**A**) LC-MS chromatogram of the methanol extract of the flowers of *S. reginae* Banks; (**B**) overlaid LC-MS chromatograms of the methanol extracts of the flowers of *S. reginae* Banks (in black) and *S. nicolai* Regel & Körn (in red); (**C**) LC-MS chromatogram of the methanol extract of *S. reginae* Banks leaves; and (**D**) overlaid LC-MS chromatograms of the methanol extracts of the leaves of *S. reginae* Banks (in black) and *S. nicolai* Regel & Körn (in red).

LC-MS/MS analysis on the flowers (Fig. 3A and B) and leaves (Fig. 3C and D) extracts of the two *Strelitzia* species led to the tentative identification of 27 components, the majority of which belonged to the phenolic acids, flavonoids, fatty acids, and high molecular weight phenolic derivatives. (Table 2).

Differences were observed between the two *Strelitzia* species (interspecies variations) and between the leaves and flowers of the same species (intraspecies variations) (Fig. 3A-D). Comparing the leaf extracts of *S. reginae* Banks and *S. nicolai* Regel & Körn (Fig. 3C and D), it was observed that phenolic and fatty acids were generally higher in *S. nicolai* Regel & Körn, whereas flavonoids and phenolic derivatives were more intense in *S. reginae* Banks. This justifies the stronger antioxidant activity observed for the leaves of *S. reginae* Banks than *S. nicolai* Regel & Körn. For the flowers, it was observed that phenolic acids were in comparable levels between the two species, whereas fatty acids were slightly higher in *S. reginae* Banks. Flavonoids were more intense in *S. nicolai* Regel & Körn flowers, whereas phenolic derivatives were higher in *S. reginae* Banks. Comparing the leaves and flowers extracts of each species, we observed stronger peak intensity signals for phenolic acids, fatty acids, and phenolic derivatives in *S. reginae* Banks flowers yet higher levels of flavonoids in *S. reginae* Banks leaves, which again justifies stronger antioxidant activity for *S. reginae* Banks leaves than the flowers. In case of *S. nicolai* Regel & Körn, the flowers displayed higher levels of phenolic acids, flavonoids, and phenolic derivatives, yet the leaves showed predominance in fatty acids. This justifies the stronger antioxidant power of *S. nicolai* Regel & Körn flowers compared to the leaves. This antioxidant activity is generally due to a synergistic effect between existing flavonoids and phenolic compounds.

#### Phenolic acids and their glycosides

Phenolic acids were the first class of compounds to be eluted in the studied extracts, however phenolic glycosides and related derivatives were the last to be eluted (Fig. 3A and B). Among the detected phenolic acids were vanillic acid and its glucoside, caffeic acid derivative, coumaroyl derivative, coumaric acid glucuronide, coniferyl aldehyde, and digalloyl glucose. Peak 1 displayed a molecular ion at *m/z* 329 and a major fragment at *m/z* 167 attributed to a vanillic acid moiety after the loss of glucose. Vanillic acid itself was similarly detected with a molecular ion peak at *m/z* 167 (peak 3). Compound 11 (t_R_ = 7 min) showed an [M-H]^−^ ion at *m/z* 415 with a base peak fragment at *m/z* 179 suggesting a caffeic acid derivative. Compound 32 exhibited a deprotonated molecular ion at *m/z* 443 with MS^2^ fragments characteristic of coumaroyl group at *m/z* 163 and *m/z* 145. Similarly, a base peak fragment at *m/z* 163 of compound 35 ([M-H]^−^ at *m/z* 339) suggesting it to be a coumaric acid glucuronide. That compound has previously been reported in the closely related plant *Morus macroura* Miq^31^. Peak 13 showed a deprotonated ion at *m/z* 177 with MS^2^ fragment at *m/z* 162 suggesting the loss of methyl group and another fragment at *m/z* 145 probably due to further loss of a hydroxy group. The compound was putatively identified as the methyl ester of coumaric acid or coniferyl aldehyde based on previous report on *Strelitzia nicolai* Regel & Körn^32^. Digalloyl glucose (peak 27, t_R_ = 16.3 min) showed similar retention time and product ions as those previously reported for the compound^33^.

#### Flavonoids

Flavonoids eluted after phenolic acids and were generally more concentrated in the extract of *S. nicolai* Regel & Körn flowers than *S. reginae* Banks (Fig. 3A and B). Peak 4 showed [M-H]^−^ at *m/z* 609 and an aglycone fragment at *m/z* 301 suggesting it to be rutin (quercetin rhamnosyl glucoside) (Fig. 3A). Quercetin-3-O-rhamnoside (peak 8, [M-H]^−^ at *m/z* 447) showed similar base peak fragment at 301 and was also detected in the flowers and leaves of *S. nicolai* Regel & Körn. A deprotonated molecular ion at *m/z* 593 (peak 6) showed MS^2^ fragment ion of the kaempferol aglycone at 285 [M-H-rhamnosyl-glucosyl]^−^ and was identified as kaempferol-3-O-rutinoside. The same fragment was observed for a peak eluting a bit earlier (t_R_ = 5.96 min) showing [M-H]^−^ peak at *m/z* 739 and was recognized as kaempferol-3-O-rutinoside-7-O-rhamnoside. Both peaks (5 and 6) were identified in the flowers and leaves of *S. reginae* Banks in accordance with what had previously been identified in the butanol fraction of the leaves of *S. nicolai* Regel & Körn^32^. Peak 7 showed a deprotonated ion at *m/z* 623 and a diagnostic base peak fragment at *m/z* 315 corresponding to the loss of rutinose (−308 Da), hence identified as isorhamnetin-3-O-rutinoside. Despite being previously identified in the leaves of *S. nicolai* Regel & Körn^32^, it was only here detected in the flowers of *S. nicolai* Regel & Körn. Other flavonoids like tricin, diosmetin, and pinobanksin were tentatively identified based on their MS^2^ fragments that matched to those reported in the literature^34–36^.

#### Fatty acids and fatty amides

Several fatty acids and their amides were identified in *Strelitzia* species including trihydroxyoctadecadienoic acid (peak 14, t_R_ = 8.0 min) which was detected in *S. reginae* Banks flowers and showing deprotonated ion peak at *m/z* 327 (Fig. 3A), trihydroxyoctadecenoic acid (peak 15, t_R_ = 9.32 min, [M-H]^−^ at *m/z* 329), 9-oxo-octadeca-10,12-dienoic acid (peak 20, t_R_ = 14.3 min, [M-H]^−^ at *m/z* 293), 9-hydroxy-10,12-octadecadienoic acid (peak 22, t_R_ = 15.4 min, [M-H]^−^ at *m/z* 295) which were all detected in *S. reginae* Banks flowers and *S. nicolai* Regel & Körn leaves. Linolenic acid was exclusively detected in *S. nicolai* Regel & Körn leaves while linoleamide and stearamide were observed in *S. reginae* Banks flowers. Fatty acids often show fragment ions attributed to the loss of CO_2_, H_2_O, and (CH_2_)_n_ in their mass spectra. Fatty acids showed comparable levels (based on their signal intensities) in *S. nicolai* Regel & Körn and *S. reginae* Banks flowers (Fig. 3B).

**Table 2:** LC-MS/MS analysis of the methanol extracts of *Strelitzia Reginae* banks and *Strelitzia Nicolai* Regel & Körn flower and leaf extracts.

Peak no.	t_*R*_ (min)	[M-H]^−^	[M + H]^+^	MS^2^	Tentatively identified metabolites	Class	Flowers		Leaves		Ref
							S. reginae	S. nicolai	S. reginae	S. nicolai	
1.	1.20	329	–	**167**	Vanillic acid glucoside	Phenolic acid glycoside	√	×	×	√	^37^
2.	1.38	293	–	**113**, 69	Methyl succinic acid-O-hexoside	Organic acid glycoside	√	×	√	×	^38^
3.	5.16	167	–	167, 151, **108**	Vanillic acid	Phenolic acid	*√*	*√*	×	×	^39,40^
4.	5.85	609	–	301, **300**, 255, 193	Rutin	Flavonoids	*√*	*√*	*√*	*√*	^32,41^
5.	5.96	739	–	**285**	Kaempferol-3‑*O*‑rutinoside‑7‑*O*‑rhamnoside		×	×	*√*	*√*	^32^
6.	6.02	593	–	285, **284**, 255, 212, 153	Kaempferol 3-O-rutinoside		×	×	*√*	*√*	^32^
7.	6.4	623	–	356, **315**, 314, 300	Isorhamnetin‑3‑*O*‑rutinoside		×	*√*	×	×	^32,42^
8.	6.54	447	–	342, **301**, 300, 270, 255, 71	Quercetin-3-O- rhamnoside		×	*√*	×	*√*	^43^
9.	6.73	–	211	175, 151, 135, 123, **109**, 95, 81, 69, 55, 43	Unidentified	–	×	×	√	×	–
10.	6.90	417	–	**243**	Unidentified	–	×	×	√	×	–
11.	7.10	415	–	**179**	Caffeic acid derivative	Phenolic acid	√	×	×	×	^44–46^
12.	7.31	329	–	**227**, 286, **151**	Tricin	Flavonoid	√	×	×	√	^47^
13.	8.08	177	–	162, 148, 145, 144, 134, 118, **116**, 105	Methyl *p*-coumarate or coniferyl aldehyde	Phenolic derivative	×	√	×	√	^32,48^
14.	8.77	327	–	281, 229, 211, 183, **171**, 167, 119, 97, 85	Trihydroxyoctadecadienoic acid	Fatty acids	√	×	×	×	^49,50^
15.	9.32	329	–	**211**, 201, 183	Trihydroxyoctadecenoic acid		√	*√*	×	√	^49,50^
16.	11.5	293	–	293, 275, 231, **183**, 58	9-Oxooctadeca-10,12-dienoic acid		√	*√*	√	√	^50,51^
17.	11.8	–	274	**274**, 256, 230, 106, 88	Unidentified	–	√	√	√	×	–
18.	12.0	–	290	290, 272, **242**, 122, 118, 104, 88, 74, 58, 43	Unidentified	–	√	×	×	×	–
19.	13.8	265	–	265, **97**	Unidentified	–	√	*√*	×	√	–
20.	14.3	445	–	**285**, **282**	Rhein or physcion hexoside	Anthracene derivative	×	×	√	√	^52,53^
21.	14.8	–	520	**184**, 125, 86	Unidentified	–	√	×	×	×	–
22.	15.4	295	–	295, **277**, 195, 179, 171	9-Hydroxy-10,12-octadecadienoic acid	Fatty acids	√	×	×	√	^54,55^
23.	15.5	–	279	279, 237, 233, 215, 201, 177, 167, 95, **81**, 67	Linolenic acid		×	×	×	√	^56^
24.	15.8	555	–	**555**, 226, 160, 149, 125	Unidentified	–	×	×	×	√	–
25.	15.8	–	496	184, 125, **104**, 86, 60	Lysophosphatidylcholine 16:0	Lipids	√	×	×	×	^57^
26.	15.8	540	–	**277**	Unidentified	–	√	×	×	×	–
27.	16.3	483	–	255, 152, **124**	Digalloyl glucose	Phenolics	×	×	√	×	^33^
28.	17.0	515	–	515, 423, 355, 295, 277, 175, 163, **145**, 89	(di)coumaroyl derivative		√	×	×	×	^48^
29.	17.2	299	–	**299**, 281, **255**	Diosmetin or chryseriol	Flavonoids	×	×	×	√	^35^
30.	17.9	517	–	425, 381, 357, 297, 175, 163, **145**, 119	(di)coumaroyl derivative	Phenolics	√	*√*	×	×	^48^
31.	18.3	–	280	263, 245, 189, 161, 133, 123, **95**	Linoleamide	Fatty amides	√	×	×	×	^16^
32.	19.2	443	–	297, 296, 163, **145**, 119	Coumaroyl derivative	Phenolic	√	×	×	×	^48^
33.	19.4	271	–	271, 253, **225**	3,5,7-Trihydroxyflavan-4-one (pinobanksin)	Flavonoids	×	×	×	√	^36^
34.	20.2	–	282	111, **97**, 83, 69, 57	Oleamide	Fatty amide	√	×	×	√	^16^
35.	20.3	339	–	339, 212, 198, 183, **163**	Coumaric acid glucuronide	Phenolic	×	√	×	×	^31^
36.	21.0	455	–	455, 422, 355, **177**, 145, 132, 118	Unidentified	–	√	×	×	×	–
37.	22.2	457	–	457, **176**, 158, 132, 117	Unidentified	–	√	*√*	×	×	–
38.	22.4	–	284	**284**, 130, 116, 102, 88, 71, 57, 43	Stearamide	Fatty amide	√	×	×	×	^16^
39.	22.8	–	639	**567**, 529, 476, 422, 236	Acetogenin derivative	Acetogenin	×	×	√	×	^58^

### In vitro antimicrobial activity

The hexane and methanol extracts of *S. reginae* Banks flowers were initially screened for their antimicrobial activities to investigate whether or not they display antimicrobial effect as for that reported on the leaves^9^. The hexane extract, at all tested concentrations (0.019–5.019 mg/ml), showed no inhibition for any of the tested strains so far (i.e., *Bacillus subtilis* ATCC 6633, *Aspergillus brasiliensis* ATCC 16404, *Enterococcus faecalis* ATCC 29212, *Pseudomonas aeruginosa* ATCC 9027, *Candida Albicans* ATCC 10231, *Escherichia coli* ATCC 8739, *Staphylococcus aureus* ATCC 6538, *Klebsiella pneumoniae* ATCC 13883, and *Salmonella enteritidis* ATCC 13078). The same case was observed for the methanol extract which was similarly inactive against all tested strains except for *Streptococcus pneumoniae* ATCC 49,619 showing a minimum inhibitory concentration (MIC) at 1.25 mg/ml.

### Assessment of antioxidant activity using DPPH and FRAP assays

We compared the antioxidant activities of the hexane and methanol extracts of *S. reginae* Banks and *S. nicolai* Regel & Körn flowers using DPPH assay. The results showed that the hexane flower extracts of both species showed comparable antioxidant effects with 8.99 ± 0.39 and 8.61 ± 0.16 µM trolox eq/mg extract for *S. reginae* Banks and *S. nicolai* Regel & Körn, respectively. On the other hand, the methanol extract of *S. reginae* Banks (609.1 ± 26.8 µM trolox eq/mg) flowers showed twice the antioxidant power of that of *S. nicolai* Regel & Körn (320.9 ± 20.9 µM trolox eq/mg). By comparing the antioxidant power of the methanol extracts of *S. reginae* Banks flowers versus the leaves, we found almost twice the antioxidant activity for the leaves than the flowers (DPPH assay showed 1157.1 ± 112.8 µM trolox eq/mg for the leaves). Similar results were obtained by FRAP assay which showed 69.2 ± 1.56 µM trolox eq/mg and 265.4 ± 17.4 µM Trolox eq/mg for the methanol extracts of *S. reginae* Banks flowers and leaves, respectively. On the other hand, it was the opposite in the case of *S. nicolai* Regel & Körn where the antioxidant activity of the methanol extract of its flowers (DPPH assay was 320.9 ± 20.9 µM trolox eq/mg and FRAP assay was 0.17 ± 0.01 µM trolox eq/mg) was twice that of its leaves (DPPH assay was 163.57 ± 2.90 µM trolox eq/mg). As the methanol leaf extract of *S. reginae* Banks showed the best antioxidant activity, further assays including ABTS, ORAC, and metal chelation were performed on that extract displaying values of 622.2 ± 20.59 µM trolox eq/mg, 3622.2 ± 234.7 µM trolox eq/mg, and 30.5 ± 2.65 μm EDTA eq/mg, respectively.

### Assessment of anti-inflammatory activity in LPS-induced RAW 264.7 cells

RAW 264.7 cells were treated with the methanol extract of *S. reginae* Banks leaves at concentrations of 10 µg/ml and 100 µg/ml. Inflammation was induced with 1 µg/ml of LPS. Nitric oxide (NO) production was assessed using the Griess assay whereas cell viability was measured using the sulforhodamine B (SRB) assay. The extract showed no cytotoxicity with cell viability of 100.1% and 98.8% for extract concentrations of 10 µg/ml and 150 µg/ml, respectively. The extract inhibited NO production in LPS-stimulated RAW 264.7 cells by 3.3% and 44.5% at concentrations of 10 µg/ml and 100 µg/ml, respectively compared to the control. This showed that the extract displayed weak or no anti-inflammatory effect.

### Target prediction and drug similarity search

As *S. reginae* Banks hexane extract is enriched in fatty acids (i.e., linoleic acid and 17-octadecynoic acid), fatty acid esters (i.e., linolenic acid methyl ester), and long chain hydrocarbons (i.e., heneicosane) whereas the hexane extract of *S. nicolai* Regel & Körn is enriched in cumene and its derivatives (i.e., pseudocumene and hexahydrocumene), target prediction using platforms such as Swiss target prediction (http://www.swisstargetprediction.ch/), and binding database (https://www.bindingdb.org/rwd/bind/chemsearch/marvin/FMCT.jsp) along with drug similarity search using therapeutic target database (https://db.idrblab.net/ttd/ttd-search/drug-similarity) showed high probabilities (i.e., of the query compounds to have that protein as target) and good similarity scores (> 0.7) for the fatty acid binding protein family, the nuclear receptor PPAR, and the trace amine associated receptor 1 (Table 3).

Cumene, which is the major component detected in *S. nicolai* Regel & Körn hexane extract, showed the highest probability for binding to trace amine associated receptor 1 (TAAR1) whose activation reduces dopamine release hence used in the treatment of psychotic disorders like schizophrenia; however, its inhibition increases dopamine levels hence used in the treatment of depression. Cumene showed intermediate similarity to phentermine (a dopamine and norepinephrine releasing agent) which is approved as an appetite depressant for treatment of obesity (Table 3). Cumene is likewise predicted to bind to the nuclear receptor, peroxisome proliferator activated receptor (PPAR-γ), which is a key regulator of glucose and lipid metabolism. Components like linoleic acid and 17-octadecynoic acid which are among the major compounds detected in the hexane extract of *S. reginae* Banks flowers, showed binding affinities to targets like adipocyte fatty acid binding protein (A-FABP or FABP4) and PPAR especially for subtype gamma. Inhibitors of FABP4 have been shown to reduce the extent of atherosclerosis and improve insulin sensitivity in genetic and/or dietary models of atherosclerosis and type 2 diabetes^59^. The activation of PPAR-γ has been reported to decrease atherosclerosis and improve insulin sensitivity^60,61^. Therefore, we tested the hexane extract of *Strelitzia reginae* Banks for its inhibitory potential of FABP4 and its agonistic effect on PPAR-γ in vitro. The hexane extract was tested for its effect on the expression of TAAR1 in vitro and for its agonistic effect on PPAR-γ as described in the next sections.

**Table 3:** Results of target prediction and drug similarity search performed on the major compounds detected in the hexane extracts of *S. reginae* banks and *S. nicolai* flowers.

Compound name/peak area % in the extract	Target	Common target name	Target class	Similarity score (on binding database)	Probability (on Swiss target prediction database)	Drug name/Tanimoto similarity
Cumene/24.1%	Trace amine-associated receptor 1	TAAR1	Family A G protein-coupled receptor	No detected targets	0.0443	Phentermine/0.806 (intermediate similarity)
	Peroxisome proliferator-activated receptor	PPAR	Nuclear receptor		0.0339	
Pseudocumene/12.4%	Acetylcholinesterase	ACHE	Hydrolase		0.1878	Melitracen/0.756 (intermediate similarity)
Hexahydro cumene/12.0%	Testis-specific androgen-binding protein	SHBG	Secreted protein		0.2483	Elemene/0.615 (remote similarity)
	Acetylcholinesterase	ACHE	Hydrolase		0.0238	
Propenyl cyclohexane/14.0%	UDP-glucuronosyl transferase 2B7	UGT2B7	Enzyme		0.0238	Elemene/0.714 (intermediate similarity)
	Nuclear receptor subfamily 1 group I member 3	NR1I3	Nuclear receptor		0.0238	
1-Ethyl-3-methylcyclo hexane/11.3%	Testis-specific androgen-binding protein	SHBG	Secreted protein		0.2483	Elemene/0.615 (remote similarity) Neramexane/0.594 (remote similarity) Memantine/0.585 (remote similarity)
	High-affinity choline transporter	SLC5A7	Electrochemical transporter		0.0339	
	Acetylcholinesterase	ACHE	Hydrolase		0.0238	
Heneicosane/18.3%	Testis-specific androgen-binding protein	SHBG	Secreted protein		0.1143	Decamethonium/0.586 (remote similarity) Mical/0.586 (remote similarity) Cetrimide/0.586 (remote similarity)
Linoleic acid/12.9%	Fatty acid binding protein adipocyte	FABP4	Fatty acid binding protein family	Fatty acid binding proteins (0.7) PPAR (0.7)	1	Epanova/0.961 (high similarity) Icosapent/0.84 (intermediate similarity)
	Peroxisome proliferator-activated receptor	PPAR	Nuclear receptor		1	
17-Octadecynoic acid/12.1%	Testis-specific androgen-binding protein	SHBG	Secreted protein	No detected targets	0.1169	ONO-2506/0.729 (intermediate similarity) CardioPET/0.711 (intermediate similarity)
	Bile acid receptor FXR	NR1H4	Nuclear receptor		0.1087	
	HMG-CoA reductase	HMGCR	Oxidoreductase		0.1005	
	Peroxisome proliferator-activated receptor	PPAR	Nuclear receptor		0.1005	
	Fatty acid binding protein adipocyte	FABP4	Fatty acid binding protein family		0.1005	

### PPAR-γ luciferase reporter assay

The hexane extracts of the flowers of *S. reginae* Banks and *S. nicolai* Regel & Körn were screened for their potential to activate peroxisome proliferator activator receptor-gamma (PPAR-γ), which plays an important role in insulin sensitivity and adipocyte differentiation, in cell-based reporter assay. Both extracts increased PPAR-γ dependent luciferase activity in a dose dependent manner. As presented in Table 4, the hexane extract of *S. reginae* Banks flowers showed better agonistic activity on PPAR-γ than *S. nicolai* Regel & Körn. Both extracts showed good PPAR-γ agonistic activities compared to the reference drug rosiglitazone. *S. reginae* Banks and *S. nicolai* Regel & Körn extracts displayed 1/4 and 1/7 the potency of rosiglitazone, respectively. Fatty acids like linoleic acid and 17-octadecynoic acid, which are enriched in *S. reginae* Banks extract, are natural ligands for PPAR-γ triggering its activation hence influencing processes such as fatty acid storage, glucose uptake, and differentiation of fat cells^62–65^. PPAR-γ activation could improve inflammatory disorders^66,67^ and enhance insulin sensitivity hence could be of therapeutic value in the treatment of type 2 diabetes and cancer^68–71^. Some natural extracts have been reported to display PPAR-γ agonistic effect due to their fatty acid content including linoleic and palmitic acids^72^.

**Table 4:** EC_50_ values of the binding of metabolites in the hexane extracts of *S. reginae* banks and *S. nicolai* Regel & Körn flowers to PPAR-γ ligand binding domain.

Samples	EC_50_ (µg/ml)
*S. reginae* Banks flowers (hexane extract)	1.203 ± 0.045*
*S. nicolai* Regel & Körn flowers (hexane extract)	1.984 ± 0.075*
Rosiglitazone	0.292 ± 0.011

### Human trace amine associated receptor-1 (TAAR1) immunoassay

**Fig. 4:**
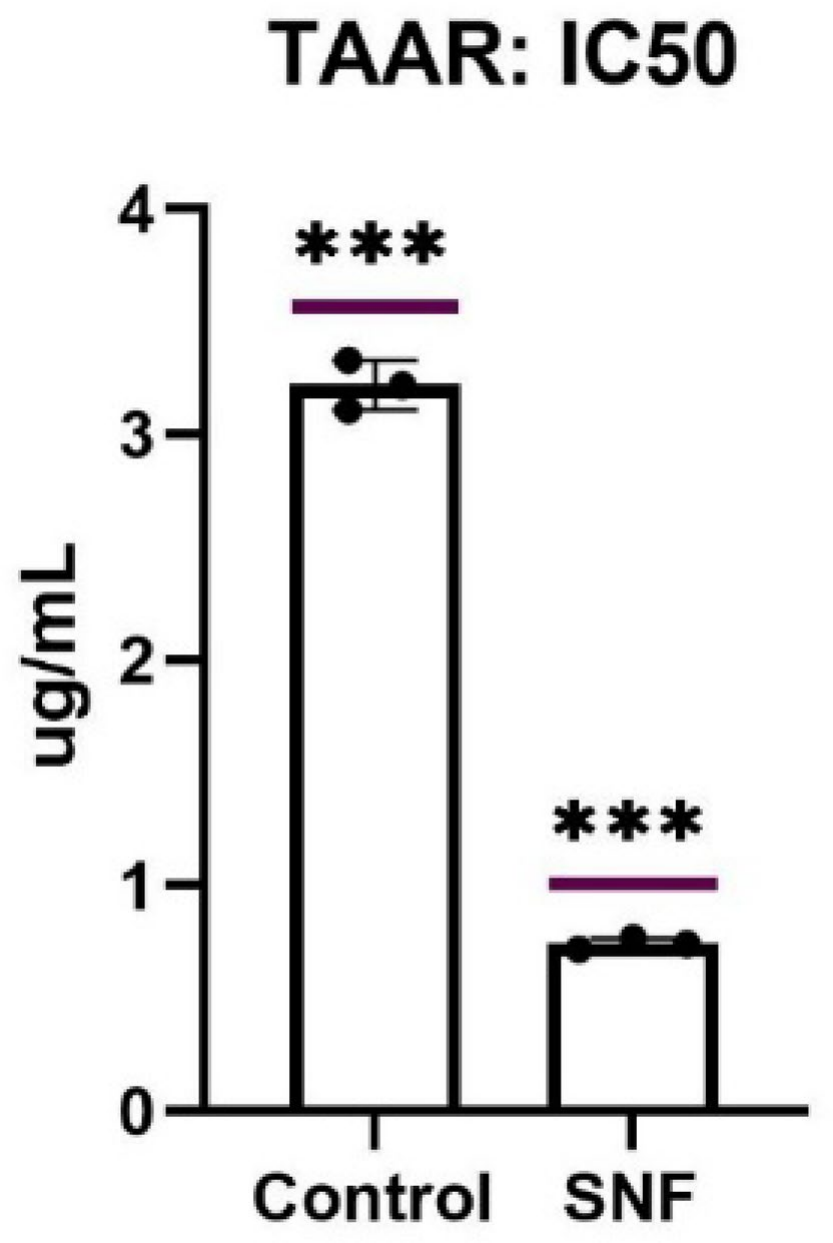
The decrease in TAAR1 levels in NCI-H810 cells treated with 10 µg/ml of the hexane extract of *S. nicolai* Regel & Körn flowers compared to the untreated cells (control). Statistical analysis was performed using one sample t-test. Data is displayed as mean ± SD. ***Significance from the control is observed at *p* < 0.05.

TAAR1 is a G-protein coupled receptor whose signaling reduces dopamine release from terminals specially in the VTA (ventral tegmental area) and decreases the firing rate of dopaminergic neurons. Agonists on TAAR1 could be used as potential candidates for treatment of schizophrenia and other psychotic disorders^73–75^, whereas blockade of TAAR1 increases the affinity of dopamine to D2 receptors hence antagonists on TAAR1 could be of potential value in the management of hypodopaminergic disorders such as depression and Parkinsonism^76^. Quantitative measurement of TAAR1 levels in NCI-H810 cells treated with 10 µg/ml of the hexane extract of *S. nicolai* Regel & Körn flowers was performed using an ELISA sandwich kit. The extract was tested due to its richness in cumene (as described in the GC-MS analysis) which shares some structural similarities to phenylethyl amines and other endogenous TAAR1 ligands of the metabolites of amino acids. Compared to the untreated cells (control) whose concentration of TAAR1 was 3.217 ± 0.11 ng/ml, treated cells showed 4x reduction in TAAR1 concentration to 0.743 ± 0.025 ng/ml (Fig. 4). These results show that cumene and other components of the hexane extract of *S. nicolai* Regel & Körn flowers might interfere with the expression or the secretion of TAAR1, hence reducing its activity.

### Fatty acid binding protein 4 (FABP4) screening assay

Fatty acid binding protein 4 (adipocyte FABP) is one of nine known cytosolic fatty acid binding proteins, that exhibit high affinity for small lipophilic molecules^77^ and are involved in the uptake and metabolism of fatty acids, in the preservation of fatty acid levels within cellular membranes, in intracellular trafficking of fatty acids, in the modulation of cell growth and differentiation as well as the modulation of enzymes involved in lipid metabolism^78^. FABP4 is highly expressed in adipocytes and is regulated by insulin, PPAR-γ agonists, and fatty acids. Therefore, inhibiting FABP4 could be of potential value in the management of diseases like atherosclerosis, diabetes, and inflammatory disorders like osteoarthritis^78,79^.

Due to the richness of the hexane extract of *S. reginae* Banks flowers with long chain hydrocarbons, fatty acids and their esters which showed good binding to FABP4 (as described in the target prediction section), we tested the potential of the extract to inhibit FABP4 experimentally using a fluorescence displacement assay. The extract showed an IC_50_ value of 1.051 ± 0.028 µg/ml which is one fifth the potency of the positive control cobimetinib (a known anticancer agent and A-FABP inhibitor^80^ with an IC_50_ value of 0.235 ± 0.015 µg/ml). The results showed that the extract displayed moderate inhibitory effect on FABP4 which might be attributed to the balance between the agonistic effect of fatty acids which are substrates on FABP4 and their ester derivatives which could act as blocking agents (by binding to the fatty acid binding site in the protein without causing its activation). It was previously reported that fatty acids are agonists on PPAR-γ whereas their esters act as antagonists^81^. Omega-3 fatty acid esters have been likewise reported to inhibit FABP4^82^ despite the agonistic effect of fatty acids themselves. In a similar way, long chain hydrocarbons such as heneicosane, pentacosane, octacosane, nonacosene, and tetratricontane, which are enriched in *S. reginae* Banks hexane extract, display the same scaffold of fatty acids yet they lack the polar carboxylate group so they might compete with the natural fatty acid ligands on FABP4.

### Molecular docking studies

In silico studies were performed to illustrate the mechanism(s) of binding between the major identified natural metabolites in *S. reginae* Banks and *S. nicolai* Regel & Körn hexane extracts with the target proteins FABP4, PPAR-γ, and TAAR1. Prior to molecular docking, the co-crystallized ligands were redocked to the active sites of the proteins using the built-in scoring functions of smina software. For FABP4, the vinardo scoring function best reproduced the experimental binding pose with a docking score of −10.27 kcal/mol and an RMSD value (between the original and redocked poses) of 0.49 Å (< 2). For TAAR1 and PPAR-γ, the vina scoring function achieved RMSD values of 0.31 and 0.55 Å with inhibitors’ binding affinities of −6.06 and − 10.71 kcal/mol, respectively. The alignments between the co-crystallized and redocked poses are shown in Fig. S1. The major natural metabolites of the two *Strelitzia* species were docked on the respective targets and their docking scores are displayed in Table 5.

**Table 5:** Molecular Docking scores of the major metabolites in the hexane extracts of *S. reginae* banks and *S. nicolai* Regel & Körn flowers on PPAR-γ, FABP4, and TAAR1.

Species	Ligand	Docking score (kcal/mol)
	FABP4	
*S. reginae* Banks	Heneicosane	−7.14
	Linoleic acid	−8.39
	17-Octadecynoic acid	−8.04
	Cobimetinib (control)	−7.89
	**TAAR1**	
*S. nicolai* Regel & Körn	Cumene	−6.09
	Pseudocumene	−6.07
	Hexahydrocumene	−5.97
	Propenylcyclohexane	−6.67
	Phenylethylamine (control)	−6.06
	**PPAR-γ**	
*S. reginae* Banks and *S. nicolai* Regel & Körn	Heneicosane	−5.62
	Linoleic acid	−6.38
	17-Octadecynoic acid	−6.14
	Cumene	−5.16
	Pseudocumene	−5.44
	Hexahydrocumene	−5.13
	Propenylcyclohexane	−5.23
	Rosiglitazone (control)	−8.88

**Fig. 5:**
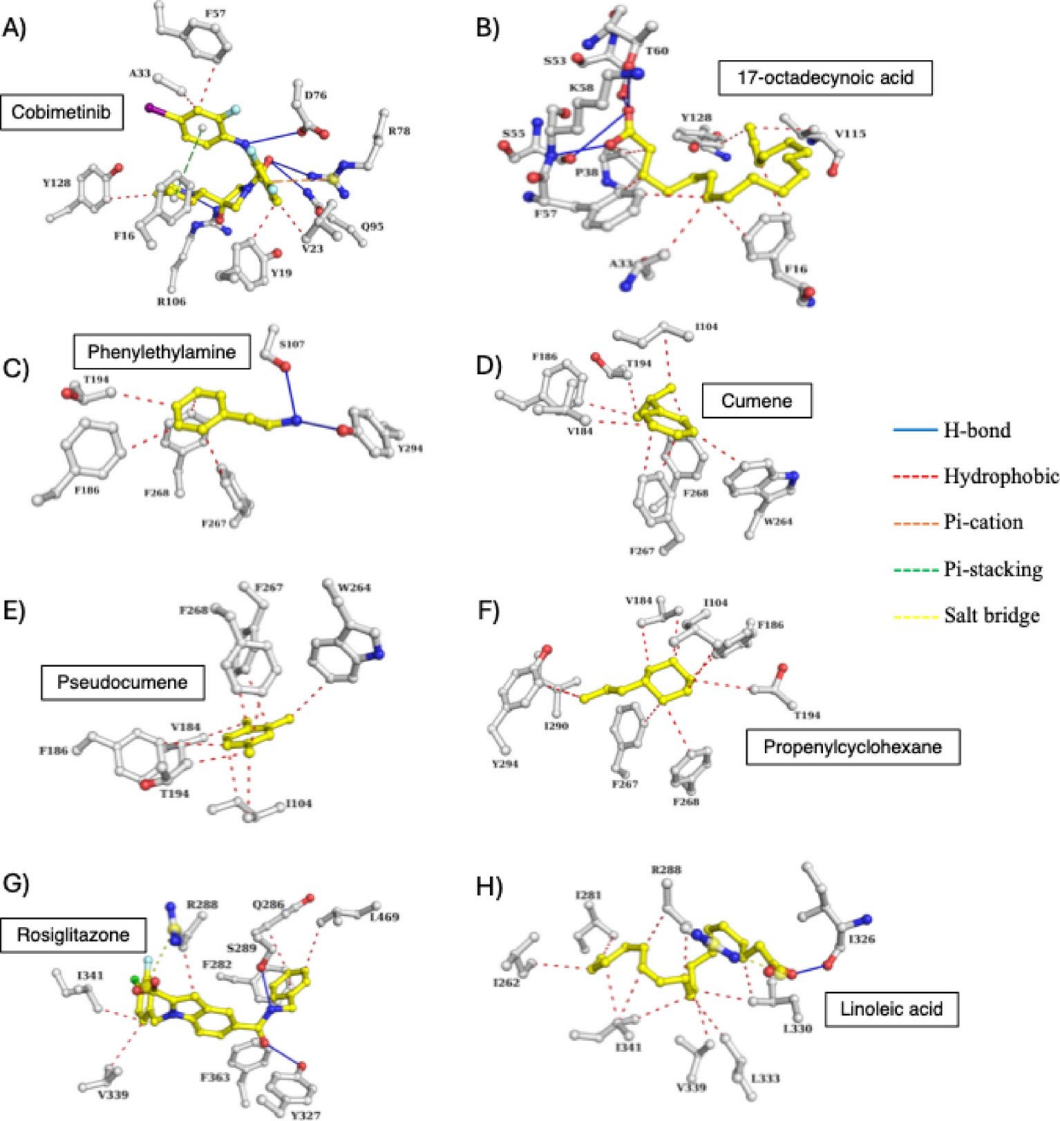
Binding modes of the reference ligands and top hit compounds against (**A**,**B**) FABP4, (**C**–**F**) TAAR1, and (**G**,**H**) PPARG. Compounds (yellow) and interacting residues (white) are shown as ball and sticks.

Cobimetinib, which is a novel inhibitor of FABP4 previously discovered by virtual screening and machine learning^80^, mediated four hydrogen bonds with D76, R78, Q95 and R106 along with hydrophobic interactions with F16, Y19, V23, A33, F57, and Y128 (Fig. 5A). A parallel π-stacking and π-cation interactions were formed with F16 and R78, respectively. On the other hand, 17-octadecynoic acid formed seven hydrophobic interactions with F16, A33, P38, F57, A115, and Y128 and four hydrogen bonds with S53, K58, and T60 (Fig. 5B).

For the TAAR1 protein, propenylcyclohexane, cumene, and pseudocumene showed higher binding affinities than the co-crystallized ligand phenylethylamine (PEA) which interacted with TAAR1 through four hydrophobic interactions (F186, T195,F267, F268) and three hydrogen bonds (S107 and Y294) (Fig. 5C). Propenylcyclohexane, cumene, and pseudocumene exclusively formed hydrophobic interactions. Propenylcyclohexane formed 10 interactions with Il104, V184, F186, T194, F267, F268, Il290, and Y294 (Fig. 5D). Cumene and pseudocumene mediated 9 interactions with Il104, V184, F186, T194, W264, F267, and F268 (Fig. 5E,F).

For PPAR-γ, rosiglitazone (a known agonist) mediated several hydrophobic interactions with F282, Q286, R288, V339, Il341, F363, and L469 along with two hydrogen bonds with S289 and Y32 and one salt bridge with R288 (Fig. 5G). None of the natural compounds showed a better docking score than rosiglitazone. Linoleic acid, with a docking score of −6.38 kcal/mol, mediated hydrophobic interactions with Il262, Il281, R288, L330, L333, V339 and Il341 and formed hydrogen bond with Il326 and salt bridge with R288 (Fig. 5H).

## Conclusion

*S. reginae* Banks and *S. nicolai* Regel & Körn are two species cultivated in Egypt. Differential GC-MS-guided profiling of the hexane extracts of their flowers revealed the abundance of saturated long chain alkanes, fatty acids and their esters in *S. reginae* Banks while cumene and its derivatives dominated the hexane extract of *S. nicolai* Regel & Körn. Comparative LC-MS/MS analysis of the methanol extracts of the flowers and leaves of both species revealed inter- and intraspecies variations in the content of phenolic acids, flavonoids, and phenolic derivatives. The hexane and methanol extracts of *S. reginae* Banks lacked antimicrobial or anti-inflammatory activities, however the methanol extract of the leaves displayed the best antioxidant activity. The hexane extract of *S. reginae* Banks moderately inhibited FABP4 (A-FABP), hence showing premise for the management of atheroscelerosis. The hexane extract of *S. nicolai* Regel & Körn reduced the levels of TAAR1 (compared to the untreated cells) so it could be of potential value for treatment of Parkinson and/or depression. The hexane extracts of the flowers of both species could activate PPAR-γ with higher potency pertained to *S. reginae* Banks, therefore the latter could be considered for further antidiabetic assays.

## Supplementary Information

Below is the link to the electronic supplementary material.

Supplementary Material 1

## Data Availability

The data that support the findings of this study are available from the corresponding author upon reasonable request.
