# Supplementary material for: Metabolomic profiling and biological evaluation demonstrate the antioxidant, PPAR-γ, TAAR1, and FABP4 modulatory potential of Strelitzia species
Source: Sci Rep. 2026 Feb 18;16:7177. doi: 10.1038/s41598-026-37621-9 (PMC12920919; doi:10.1038/s41598-026-37621-9)

**Supplementary File**

**Antioxidant, PPAR-γ activation, and TAAR1/FABP4 inhibition by *Strelitzia reginae* and *Strelitzia nicolai*: A study supported by GC-MS, ESI-MS/MS, and docking**


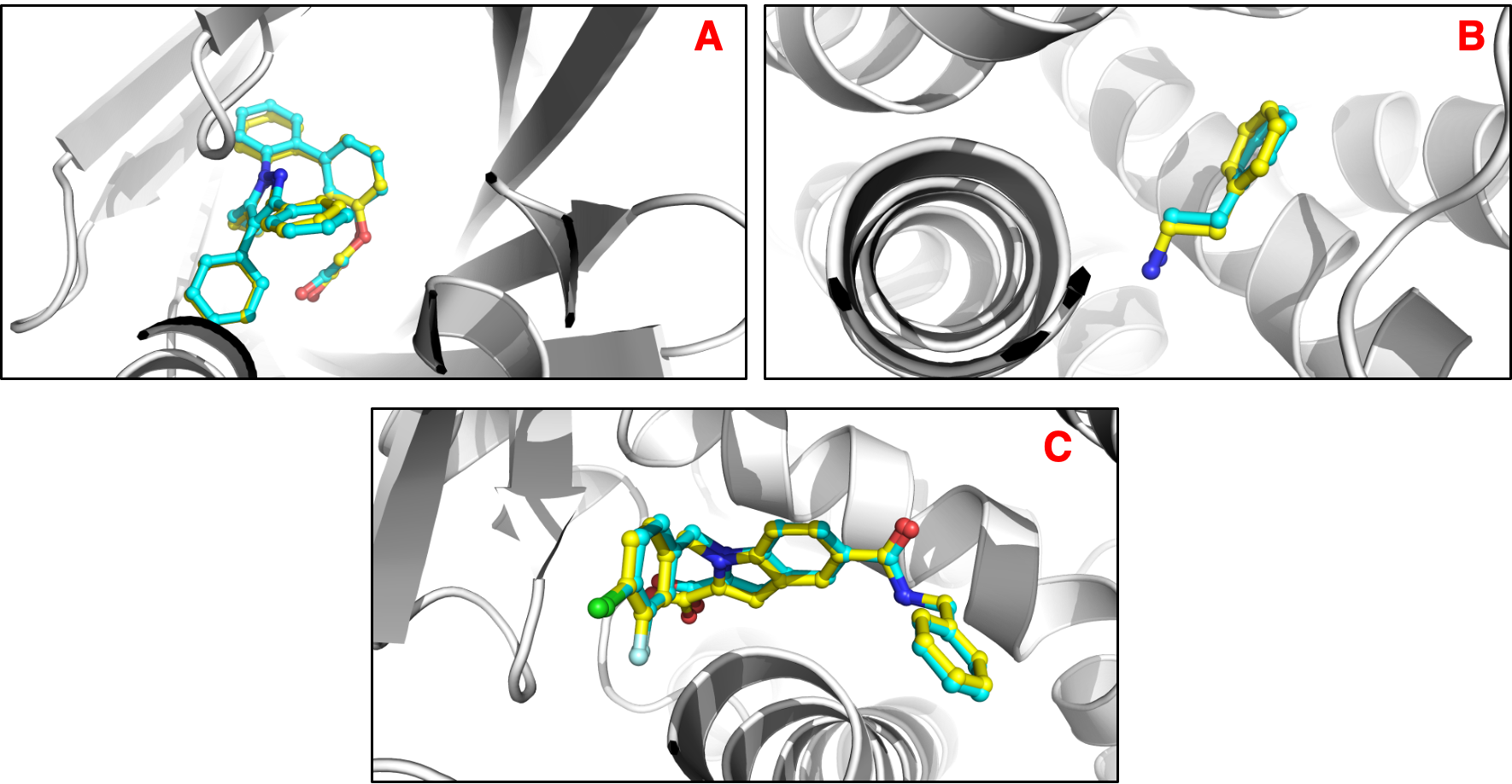


**Fig. S1. Alignment between the co-crystallized and redocked ligands for (A) FABP4, (B) TAAR1, and (C) PPAR-γ .** Experimental and redocked poses are shown as yellow and cyan sticks, respectively.


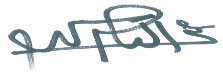

Supplement: Supplementary file 1 — Supplementary Material 1 [file 41598_2026_37621_MOESM1_ESM.docx]
